# Equine Pergolide Toxicity: A Case Series

**DOI:** 10.7759/cureus.64265

**Published:** 2024-07-10

**Authors:** Natalie E Ebeling-Koning, John T Fowler, John D DelBianco, Ryan M Surmaitis

**Affiliations:** 1 Department of Emergency and Hospital Medicine, Lehigh Valley Health Network/University of South Florida (USF) Morsani College of Medicine, Allentown, USA

**Keywords:** equine, dopamine receptor agonist, unintentional ingestion, veterinary medication exposure, pergolide toxicity

## Abstract

Veterinary medication exposure may result in human toxicity, with approximately 6,000 exposures to veterinary-only medications reported to poison centers in 2022. There is a paucity of literature on the management of poisoned patients secondary to pharmaceuticals intended for equine use. Pergolide is a dopamine and serotonin receptor agonist and is currently approved to treat equine Cushing’s disease. It was previously approved in the United States (US) to treat Parkinson’s disease in humans; however, it was withdrawn from the market in 2007 due to its association with valvular heart disease. We report two cases of pergolide toxicity in horse owners following unintentional ingestions. Both patients experienced similar clinical presentations resulting from their unintentional pergolide ingestions. Veterinary medication ingestion presents a unique challenge to clinicians as the drug may have limited human toxicity data and/or recommended animal dosing may differ greatly from human dosing. Case reports of human toxicity may assist with anticipating the clinical course and guiding medical decision-making.

## Introduction

Humans may be exposed to an array of veterinary medications, some of which are not approved for human use and may be unfamiliar to clinicians. Approximately 6,000 exposures to veterinary-only medications were reported to the United States (US) poison centers in 2022 [1]. Pergolide is an ergot-derived dopamine and serotonin agonist currently approved in the US for pituitary pars intermedia dysfunction (PPID), also known as equine Cushing’s disease [2,3]. It was previously used to treat Parkinson’s disease in humans but was withdrawn from the US and Canadian markets in 2007 due to its association with the development of cardiac valvular regurgitation affecting the tricuspid, mitral, and aortic valves [4-8]. We report two cases of pergolide toxicity following unintentional ingestions.

This case series was presented, in part, at the North American Congress of Clinical Toxicology on October 1, 2023, and presented at the Pennsylvania College of Emergency Physicians Scientific Assembly on May 3, 2024.

## Case presentation

Case 1

**Figure 1:**
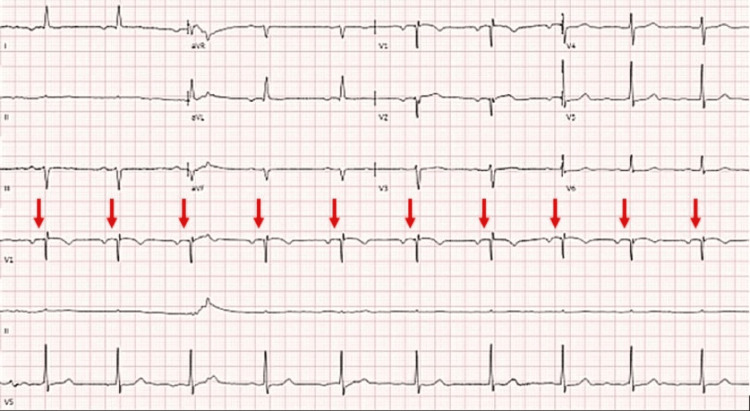
Patient 1 ECG with arrows showing sinus rhythm with first-degree AV block and no ectopy ECG: electrocardiogram; AV: atrioventricular

A 75-year-old female with a history of anxiety on citalopram presented to the emergency department (ED) after unintentional ingestion of pergolide 0.5 mg that had been placed into a fig cookie and intended for a horse. Shortly after ingestion, bystanders described an acute episode of neuromuscular rigidity without loss of consciousness. She experienced vomiting en route to the ED. On arrival, she was alert and oriented but was described as drowsy with refractory nausea and dizziness. Her initial vital signs were: temperature of 96.2ºF (35.6ºC), heart rate (HR) of 61 beats per minute, respiratory rate (RR) of 14 breaths per minute, blood pressure (BP) of 117/63 mmHg, and oxygen saturation of 96% on room air. She was administered ondansetron 4 mg intravenously (IV) and 1 L of normal saline (NS). An electrocardiogram (ECG) showed sinus rhythm with first-degree atrioventricular (AV) block (Figure 1). There was no previous ECG available for comparison. 

Complete blood count (CBC) and comprehensive metabolic panel (CMP) were unremarkable. Medical toxicology was contacted by telephone and recommended ongoing hemodynamic monitoring and a six-hour observation period in the ED. Approximately five hours after arrival, she experienced orthostatic hypotension with a precipitous drop in BP to 80/50 mmHg upon sitting up. She noted ongoing dizziness and nausea, so she was administered prochlorperazine 5 mg IV and 1 L of lactated Ringer’s solution (LR). After nine hours in the ED, her symptoms had resolved, and her vital signs normalized. She was discharged home. Comprehensive urine drug testing with liquid chromatography/quadrupole time-of-flight mass spectroscopy (LC/QTOF/MS) was performed to identify any co-ingestants and detected only caffeine and her prescribed citalopram. Pergolide was not included in this assay and serum concentration was not available.

Case 2

**Figure 2:**
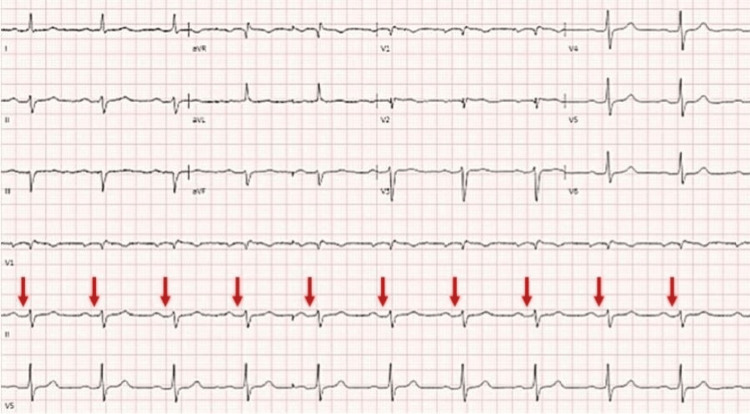
Patient 2 ECG with arrows showing sinus rhythm with first-degree AV block ECG: electrocardiogram; AV: atrioventricular

A 67-year-old female with a history of meningioma and chronic mandibular osteomyelitis presented to the ED via emergency medical services (EMS) following an unintentional ingestion of pergolide 5 mg that was intended for her horse. She reported that she had laid the pill out on the counter and subsequently mistook the horse’s medication for her own. Within 15 minutes of ingestion, she developed nausea, vomiting, chills, diaphoresis, and lightheadedness. Her BP measured by EMS was 84/46 mmHg, and she was administered 1 L of NS en route to the ED. On arrival, her BP had improved to 101/73 mmHg. She was afebrile with a temperature of 96.7ºF (35.9ºC), HR was 63 beats per minute, RR was 14 breaths per minute, and oxygen saturation was 98% on room air. Her Glasgow Coma Scale was 15 and point-of-care glucose was 114 mg/dL (ref: 65-99 mg/dL). She was administered ondansetron 4 mg IV for persistent nausea and 1 L of LR. ECG demonstrated sinus rhythm with first-degree AV block, which was similar to a previous ECG (Figure 2).

CBC was unremarkable, and CMP demonstrated mild hypokalemia with potassium of 3.4 mmol/L (ref: 3.5-5.2 mmol/L), hyperchloremia with chloride of 110 mmol/L (ref: 100-109 mmol/L), and hypocalcemia with calcium of 8.1 mg/dL (ref: 8.5-10.1 mg/dL). Acetaminophen and salicylate concentrations were undetectable. Point-of-care cardiac ultrasound showed normal systolic function, no clinically significant effusion, and a collapsed inferior vena cava. The medical toxicology service performed a bedside consultation on the patient in the ED and recommended continued supportive care with a plan for discharge once her symptoms resolved. Due to refractory nausea and inability to tolerate oral intake, she was given fosaprepitant 150 mg IV, an additional 1 L of LR, and admitted to the hospital. The patient’s symptoms were resolved by the next morning, and she was discharged home. Comprehensive urine drug testing with LC/QTOF/MS was performed and detected only caffeine.

## Discussion

Equine pergolide toxicity in humans has not been previously reported. Both of our patients experienced similar clinical effects from their unintentional pergolide ingestions. Toxicity resulted in mild sedation in one patient and hypotension, nausea, and vomiting in both. Both patients responded well to supportive care with IV fluids and antiemetics. The 75-year-old patient’s symptoms resolved while in the ED and the 67-year-old patient recovered within 24 hours.

Although pergolide is no longer approved for human use in the US, it is widely available for equine use. It is estimated that there are 1,013,746 horse owners in the US and equine PPID could affect up to 25% of older horses. Pergolide is the first-line pharmacotherapy for this condition [3,9]. Pergolide may be ingested by people either accidentally, as described here, or intentionally. It is important for clinicians, especially those practicing in rural areas where pergolide use may be more prevalent, to understand the clinical effects and management of toxicity.

Both the therapeutic and adverse effects of pergolide arise primarily from its action as a dopamine receptor agonist. In horses with PPID, decreased hypothalamic dopamine leads to disinhibition of the pars intermedia of the pituitary gland and over-secretion of adrenocorticotropin and other hormones. Pergolide restores pituitary inhibition in cases of horses with PPID [3,10]. Dopamine agonists are similarly advantageous in the treatment of both Parkinson’s disease and restless leg syndrome (RLS) [11,12]. Pergolide and other dopamine agonists, including pramipexole and ropinirole, are known to cause orthostatic hypotension, even at recommended doses. In one study of patients with Parkinson’s disease, 34% developed orthostatic hypotension after the initiation of pergolide, pramipexole, or ropinirole [13]. The hypotension associated with dopamine agonists is attributed to peripheral vasodilation [13,14]. Therapeutic use of ropinirole for RLS was associated with orthostatic hypotension and recurrent falls in one 71-year-old patient. The patient’s symptoms resolved after the medication was stopped [15]. A 59-year-old patient had dizziness with positional changes after a polypharmacy overdose that included pramipexole, which he was taking for RLS. The dizziness resolved with supportive care, including IV fluids [16]. Nausea and somnolence are also well-described adverse effects of this drug class with therapeutic dosing [14]. One meta-analysis found significantly more RLS patients receiving ropinirole experienced nausea and vomiting compared to placebo, with 37.2% of those taking ropinirole having nausea [17]. Our patients’ signs and symptoms mirror the adverse effects of dopamine agonists described in the literature. General recommendations for the management of hypotension in patients taking dopamine agonists include compression stockings and consideration of medication to improve symptoms, such as fludrocortisone or midodrine [13,14]. In the acute management of hypotension from xenobiotic exposure, isotonic IV fluid boluses are the first-line therapy. Vasopressors can be used if the patient’s blood pressure fails to improve with IV fluids alone [18].

Previously recommended human dosing of pergolide started at 0.05 mg daily and was carefully titrated to achieve an average total daily dose of 3 mg [19]. Our patients received doses that were 10 and 100 times higher, respectively, than the recommended starting dose, with the patient who ingested the higher dose experiencing more protracted symptoms and requiring a brief hospitalization. Pharmacokinetics of pergolide demonstrate that the peak plasma concentration occurs within two to three hours of ingestion, and the half-life is approximately 21 hours [20]. Given the paucity of toxicokinetic data, the treatment of any patient after an overdose must be individualized and continued for as long as he or she remains symptomatic.

## Conclusions

Pergolide is commonly used in the US for the treatment of equine PPID and may be a potential source for human exposure. The patients in our case series both presented the same clinical symptoms. Veterinary medication ingestions are uniquely challenging to clinicians because of unfamiliarity with the toxicologic effects of these medications. Physicians' lack of familiarity with the toxicological effects of veterinary drugs, such as pergolide, may make clinical management difficult. This challenge is compounded because toxicological effects can differ significantly from those seen with human medications. Case reports of pergolide human toxicity may aid in anticipating the clinical course and guiding medical decision-making.
